# Influence of Cutting-Edge Micro-Geometry on Material Separation and Minimum Cutting Thickness in the Turning of 304 Stainless Steel

**DOI:** 10.3390/ma19030591

**Published:** 2026-02-03

**Authors:** Zichuan Zou, Yang Xin, Chengsong Ma

**Affiliations:** School of Mechanical & Electrial Engineering, Guizhou Normal University, Guiyang 550025, China; xinyang2805027622@163.com (Y.X.); 13464811023@163.com (C.M.)

**Keywords:** minimum cutting thickness, finite element, 304 stainless steel, dislocation density

## Abstract

The micro-geometry of the cutting edge plays a crucial role in material flow ahead of the cutting edge and chip formation, primarily influencing chip formation mechanisms and the minimum cutting thickness. In the context of turning 304 stainless steel, however, existing research still lacks a unified quantitative framework linking “cutting edge micro-geometry—material separation behavior (separation point/minimum uncut chip thickness)—microstructural evolution of the machined surface.” This gap hampers mechanistic optimization design aimed at enhancing machining quality. This study examines the turning of 304 stainless steel by integrating analytical modeling, finite element simulation, and experimental validation to develop a predictive model for minimum cutting thickness. It analyzes the effects of tool nose radius and asymmetric edge morphology, and a microstructure evolution prediction subroutine is developed based on dislocation density theory. The results indicate that the minimum cutting thickness exhibits a positive correlation with the tool nose radius, and their ratio remains stable within the range of 0.25 to 0.30. Under asymmetric edge conditions, the minimum cutting thickness initially increases and then decreases as the K-factor varies. The developed subroutine, based on the dislocation density model, enables accurate prediction of dislocation density, grain size, and microhardness in the machined surface layer. Among the factors considered, the tool nose radius demonstrates the most pronounced influence on microstructure evolution. This research provides theoretical support and a technical reference for optimizing cutting-edge design and enhancing the machining quality of 304 stainless steel.

## 1. Introduction

Metal cutting is a core manufacturing process for shaping materials and ensuring precision. The cutting edge serves as the direct interface during cutting, and its micro-geometry—including features such as the tool nose radius and edge morphology—directly affects material flow, chip formation, cutting force distribution, and the quality of the machined surface [1]. Under the precise conditions of micro-cutting, the conventional assumption of an ideally sharp cutting edge becomes invalid. The tool nose radius is comparable in scale to the cutting thickness, which results in a negative effective rake angle. This leads to a transition in the material removal mechanism from one dominated by shear to a coupled shear-ploughing process [2]. Such an effect is especially significant when machining difficult-to-machine materials. Due to its excellent corrosion resistance, toughness, and workability, 304 stainless steel is widely used in various industrial sectors, including chemical equipment, medical devices, food processing machinery, and aerospace components [3]. However, during machining, it is prone to issues such as work hardening and high sensitivity to surface quality. Therefore, the role of cutting-edge micro-geometry in regulating the material separation behavior of 304 stainless steel demands special attention.

The minimum cutting thickness is a critical parameter for evaluating material machinability, defined as the smallest removable material unit that can produce a continuous chip. Its determination is inherently linked to the position of the material separation point [4]. This separation point constitutes the critical location where the material flow ahead of the tool splits. The material above this point forms the chip, whereas the material below it is extruded and ploughed, ultimately forming the machined surface [5]. In initial studies, Malekian et al. [6] developed an analytical model relating the minimum cutting thickness to the tool edge radius and friction coefficient, based on the minimum energy principle and the infinite shear strain method. Through finite element simulation, Lai et al. [7] determined that the minimum cutting thickness for OFHC copper was 0.25 times the edge radius, thereby confirming the dominant influence of the edge radius. With further investigation, Wan et al. [8] indicated that the stability of the separation point is affected by the dead metal zone (DMZ), the formation of which is closely tied to the edge geometry and cutting parameters. Considering unsteady cutting conditions with chip fracture, Song et al. [9] categorized the dead metal zone into four types, which further refined the mechanistic relationship between the separation point and the minimum cutting thickness. While these studies have established a foundation for understanding the fundamental nature of material separation, the majority have concentrated on symmetric edge geometries, leaving a lack of systematic analysis regarding the effects of asymmetric edge morphology.

The diversity of cutting-edge micro-geometry constitutes a significant factor influencing cutting performance. In addition to the tool nose radius, asymmetric edges—such as chamfered edges and waterfall-type edges—are extensively employed due to their ability to increase edge strength and suppress vibration [10]. Based on the finite element method (FEM) simulation, Woon et al. [11] established that the ratio of the edge radius to the cutting thickness dictates the chip formation mechanism, and material removal is primarily through extrusion when this ratio is below 1. Research by Hosseini et al. [12] showed that asymmetric edges change the location and dimensions of the dead metal zone, which consequently affects cutting forces and the quality of the machined surface. Denkena et al. [13] introduced the K-factor as a characterization method for describing asymmetric edge morphology. Nevertheless, the quantitative relationship governing how the K-factor affects the material separation point and the minimum cutting thickness has not been fully elucidated, indicating a need for further research in this area.

The evolution of the microstructure in a machined surface directly reflects the coupled thermo-mechanical effects present during cutting. Dislocation density, a fundamental physical parameter characterizing the extent of plastic deformation, provides the crucial link between macroscopic cutting conditions and microscopic material properties. Estrin et al. [14] developed a constitutive model founded on dislocation density, which facilitates the description of material hardening behavior under large strains. Jin et al. [15] integrated a dislocation density-based model into finite element analysis software, enabling the prediction of grain refinement phenomena during the cutting process. Nonetheless, prevailing research often overlooks the coupled effect of cutting-edge micro-geometry on microstructure evolution. This omission has hindered the establishment of quantitative relationships connecting edge geometry parameters, material separation behavior, and the resulting microstructure. Consequently, it remains challenging to fulfill the demands of precision machining for precise control over surface integrity.

In recent years, research on the coupling among cutting-edge micro-geometry, material flow, separation, and surface integrity has gradually shifted from the traditional equivalent edge radius to the quantitative characterization of real micro-geometry and the mechanisms governing material flow. It has been pointed out that the DMZ—separation point migration—minimum uncut chip thickness variation is one of the key factors in explaining the size effect in micro-scale cutting [16,17]. For determining material separation criteria and the minimum uncut chip thickness, various studies have emerged that incorporate the separation point and DMZ into models [11,18,19]. Furthermore, at a smaller scale, the material removal mechanisms are closely linked to the fundamental nature of the separation point and the minimum uncut chip thickness, which are often closely linked to local elastoplastic transition, dislocation accumulation, and crack initiation ahead of the cutting edge. Relevant studies show that the minimum uncut chip thickness and tool geometry can alter the dominant material-removal mechanism and the morphology of subsurface damage, thereby influencing surface quality and deformation-layer characteristics [20,21]. At the engineering materials level, determining the minimum uncut chip thickness for materials like Ti6Al4V and quantifying its relationship with the edge radius also provides a reference method for comparing how edge scale affects separation behavior across different material systems [22]. Therefore, for the turning process of 304 stainless steel, it is necessary to build on existing research and establish a clearer coupling between the quantitative characterization of asymmetric cutting-edge micro-geometry and the material separation point location, minimum uncut chip thickness, and microstructural evolution. This will enable the prediction and control of machined surface quality.

In summary, existing research on the mechanisms linking asymmetric edge morphology to the material separation point and minimum cutting thickness is not systematic, and a coupled model capable of predicting the effects of cutting-edge micro-geometry on microstructure evolution is lacking. Although numerous analytical and numerical models have been developed to describe minimum cutting thickness and dead metal zone formation, most of them are primarily evaluated based on macroscopic responses such as cutting forces or chip morphology. The influence of asymmetric cutting-edge micro-geometry on machining-induced microstructural evolution is usually treated implicitly or neglected. Consequently, existing models provide limited guidance for cutting-edge design when surface integrity and subsurface microstructure are of primary concern. The purpose of this research is a comprehensive study of the turning process of 304 stainless steel with an emphasis on the effect of micro-geometry of the cutting tool on the quality of processing. To achieve this purpose, the research is aimed at solving the following tasks: to develop theoretical models; to create software; and develop experimental data verification. Thus, the research is aimed at creating an integrated approach to optimizing the turning process of stainless steel, taking into account the micro-geometric parameters of the cutting tool.

## 2. Minimum Cutting Thickness

### 2.1. Definition of Minimum Cutting Thickness

**Figure 1:**
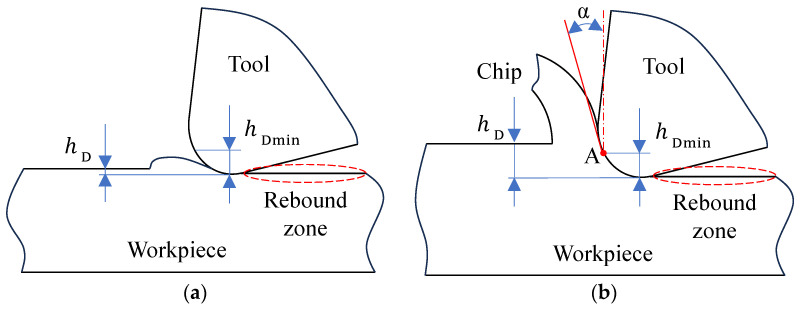
Schematic of the minimum cutting thickness. (**a**) No chip formation; (**b**) chip formation.

To investigate the elastic-plastic deformation of material ahead of the tool edge and the chip fracture formation process, analysis of the metal cutting model must be conducted at the mesoscopic scale. At this level of detail, the cutting-edge micro-geometry cannot be treated as ideally sharp, and the impact of the tool nose radius on the cutting process must be taken into account. Within the metal cutting process, the material separation point is identified as the location where material rigidity is entirely lost. The minimum cutting thickness ($h_{Dmin}$) is defined as the distance from this separation point to the generated machined surface, representing the smallest removable material unit that can produce a continuous chip. A schematic of the minimum cutting thickness is presented in Figure 1. Owing to the presence of the tool nose radius, a negative effective rake angle condition arises when the depth of cut is decreased to a value smaller than the nose radius itself. In this regime, only a minor segment of the tool nose arc actively participates in the cutting process.

If the actual depth of cut is less than the minimum cutting thickness, as depicted in Figure 1a, the workpiece material is predominantly acted upon by ploughing forces. The material layer intended for removal undergoes merely elastic deformation, preventing the formation of a continuous chip; any displaced material forms through a pushing/extrusion mode. In contrast, when the actual depth of cut exceeds the minimum cutting thickness, as illustrated in Figure 1b, shearing forces become dominant in the workpiece material, initiating the generation of a continuous chip. For AISI 304 stainless steel, the minimum uncut chip thickness is not a fixed value; it depends critically on the tool edge radius, frictional contact conditions, and operational parameters. Given the inevitable presence of an edge radius on the cutting tool and the significant influence of edge bluntness and separation point migration on material separation at small uncut chip thicknesses, the configuration that accounts for the edge radius effect—illustrated in Figure 1b—provides a more accurate representation of the machining conditions and material behavior investigated in this work. Figure 1a primarily serves as a schematic of an ideal reference.

It is generally recognized that a material separation point exists ahead of the cutting edge, identified as point A in Figure 1b. The workpiece material above this point plastically deforms under the tool’s extrusion, flows along the rake face, and thereby forms the chip. Conversely, the material below the separation point is affected by extrusion and friction, making contact with the tool’s flank face. Following elastic recovery, this material constitutes the machined surface. The distance from the separation point to this machined surface defines the minimum cutting thickness. Given the complexity and high cost of direct experimental observation, this study establishes both analytical and finite element models for the minimum cutting thickness. These models are used to analyze how variations in the tool nose radius and friction coefficient influence the minimum cutting thickness, material separation behavior, and flow patterns.

### 2.2. Analytical Model Solution

This section presents an analytical model founded on the theoretical framework of classical orthogonal cutting mechanics. The model’s fundamental assumptions and derivation logic stem from the shear-dominant cutting model proposed by Merchant [23], and it further extends the analytical cutting theories advanced by researchers, including Oxley and Childs [24,25]. By employing mechanical equilibrium and stress decomposition to characterize the stress state in the tool-workpiece contact zone, the model is well-suited for analyzing plastic deformation during the cutting of ductile metals.

**Figure 2:**
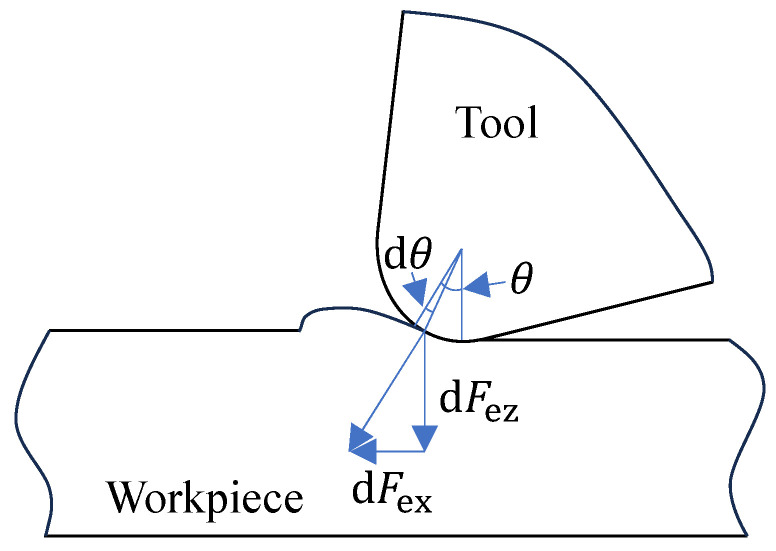
Force model of the elastic zone.

Furthermore, to incorporate the impact of realistic cutting-edge micro-geometry on material separation behavior, the model integrates theories concerning the edge effect and the minimum uncut chip thickness. These theories are widely recognized for describing the critical conditions governing the transition from a shear-based chip formation mechanism to one dominated by ploughing and extrusion [26,27]. Therefore, the analytical model developed herein is not reliant on empirical postulation; instead, it represents a deliberate application and extension grounded in established principles of cutting mechanics and material separation theory. When the actual depth of cut is below the minimum cutting thickness, the workpiece deforms elastically without plastic flow. A force model depicting a differential element within this elastic zone is presented in Figure 2.

The normal and tangential force components acting on the differential element are given by Equation (1).

(1) $$\{\begin{array}{c} dF_{ex}=p_{e}rd\theta sin\theta +\mu p_{e}rd\theta cos\theta \\ dF_{ez}=p_{e}rd\theta cos\theta -\mu p_{e}rd\theta sin\theta \end{array}$$

where $F_{ex}$ denotes the normal force component, $F_{ez}$ represents the tangential force component, $p_{e}$ is the normal stress acting on the tool nose radius within the elastic region, r signifies the tool edge radius, μ  is the friction coefficient at the tool-workpiece interface. θ is the central angle corresponding to the tool nose arc. Consequently, Equation (2) is derived as follows:

(2) $$\frac{dF_{ex}}{dF_{ez}}=\frac{p_{e}rd\theta \sqrt{\left(1+\mu^{2}\right)}sin\left(\theta +\beta_{e}\right)}{p_{e}rd\theta \sqrt{\left(1+\mu^{2}\right)}cos\left(\theta +\beta_{e}\right)}=\tan\left(\theta +\beta_{e}\right)$$

where $\beta_{e}$ denotes the friction angle at the tool-workpiece interface within the elastic region, which is governed by Equation (3):

(3) $$\mu =\tan\beta_{e}$$

**Figure 3:**
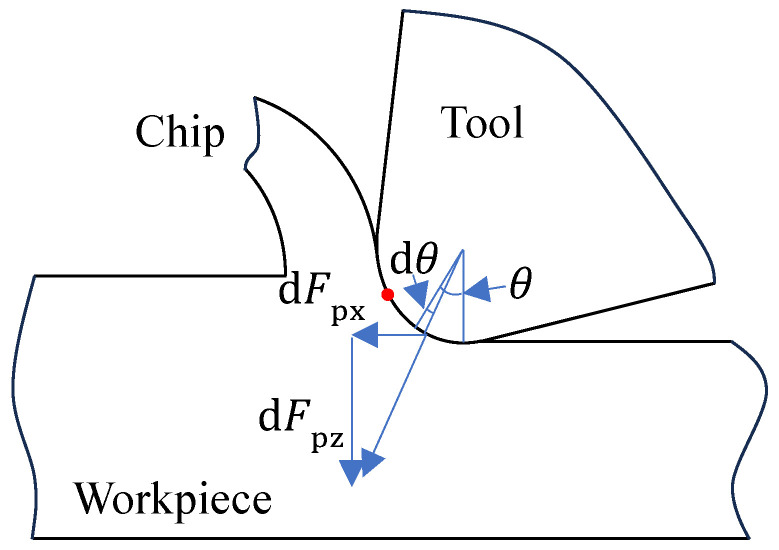
Force model for the plastic region. The red dot represents the location of the separation point.

When the actual depth of cut exceeds the minimum cutting thickness, plastic deformation becomes the dominant mode of deformation in the workpiece. The corresponding force model for a differential element located in the plastic region is presented in Figure 3.

The principal cutting force and cutting resistance for the differential element are expressed by Equation (4).

(4) $$\{\begin{array}{c} dF_{px}=\frac{\tau_{s}wcos\left(\beta_{p}-\gamma_{o}\right)}{sin\phi cos\left(\phi +\beta_{p}-\gamma_{o}\right)}dt\\ dF_{pz}=\frac{\tau_{s}wsin\left(\beta_{p}-\gamma_{o}\right)}{sin\phi cos\left(\phi +\beta_{p}-\gamma_{o}\right)}dt \end{array}$$

where $F_{px}$ represents the principal cutting force, $F_{pz}$ denotes the cutting resistance, $\tau_{s}$ is the material shear strength, w is the tool width, $\beta_{p}$ is the friction angle at the tool-chip interface, $\gamma_{o}$ is the tool rake angle, and ϕ is the shear angle. Given the relationship expressed in Equation (5),

(5) $$\{\begin{array}{c} dt=rsin\theta d\theta \\ \gamma_{o}=-\frac{\pi }{2}+2\beta_{p}+\theta \end{array}$$

Substituting Equation (5) into Equation (4) leads to

(6) $$\{\begin{array}{c} \;dF_{px}=\frac{\tau_{s}wrsin\theta sin\left(\beta_{p}+\theta \right)}{-sin\phi sin\;h\left(\phi -\beta_{p}-\theta \right)}d\theta \\ dF_{pz}=\frac{\tau_{s}wrsin\theta cos\left(\beta_{p}+\theta \right)}{-sin\phi sin\left(\phi -\beta_{p}-\theta \right)}d\theta \end{array}$$

Consequently, the ratio between the principal cutting force and the cutting resistance can be obtained from Equation (6) as

(7) $$\frac{dF_{px}}{dF_{pz}}=\frac{sin\left(\beta_{p}+\theta \right)}{cos\left(\beta_{p}+\theta \right)}=tan\left(\beta_{p}+\theta \right)$$

**Figure 4:**
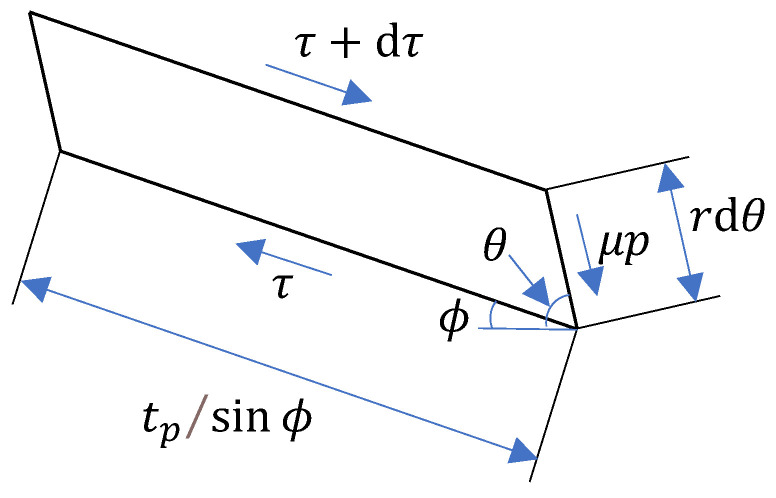
Force analysis at the stagnation point.

A comparison of Equations (6) and (7) reveals that the expression for the ratio of the normal to the tangential force component is the same for both elastic and plastic deformation. However, the numerical value of this ratio differs because the friction coefficients in the two regions are not identical. It is generally accepted that the tool nose radius induces the formation of a dead metal zone (or stagnant zone) with zero material flow velocity ahead of the cutting edge. Assuming the shear angle at the stagnation (or separation) point A equals the material’s stagnation angle, the analysis of a differential element at this location is illustrated in Figure 4. Applying force equilibrium principles to this element yields the force equation at point A, expressed as Equation (8).

(8) $$\frac{dF_{ex}}{dF_{ez}}=\frac{d\tau \;rd\theta \;cos\theta_{c}}{d\tau \;rd\theta \;sin\theta_{c}}$$

Consequently, the mathematical relationship described by Equation (9) is derived.

(9) $$\{\begin{array}{c} tan\left(\beta_{e}+\theta_{c}\right)=cot\theta_{c}\\ tan\left(\beta_{p}+\theta_{c}\right)=cot\theta_{c} \end{array}$$

Based on the maximum shear stress theory (the third strength theory), material failure is defined to occur when the maximum shear stress under a complex stress state reaches the material’s yield strength under uniaxial tension [28]. This maximum shear stress acts in a direction at 45° to the resultant cutting force. At this orientation, the feed force and the back force are equal, and the direction of cutting motion aligns with the direction of the maximum shear stress at the separation point A. The material located above point A flows along the tool rake face to form the chip. In contrast, the material below point A is subjected to extrusion and ploughing against the tool flank face, thereby generating the machined surface. The shear angle associated with point A can therefore be expressed as:

(10) $$\theta =\frac{\pi }{4}-\frac{\beta }{2}$$

The analytical model for the minimum cutting thickness is ultimately obtained as follows:

(11) $$h_{Dmin}=r\left(1-\cos\theta_{c}\right)=r\left[1-cos\left(\frac{\pi }{4}-\frac{\beta }{2}\right)\right]=r\left[1-\cos\left(\frac{\pi }{4}-\frac{\arctan\mu }{2}\right)\right]$$

**Figure 5:**
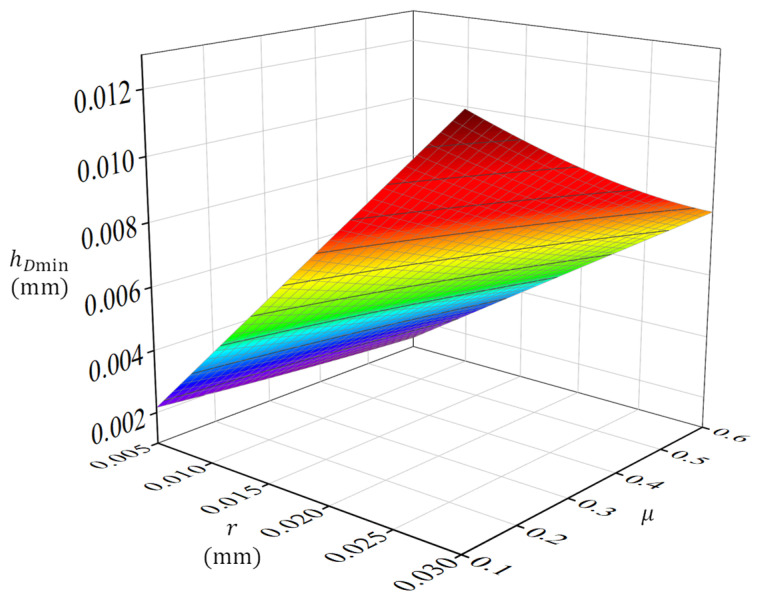
Response surface plot (Increasingly from blue to green to yellow to red).

In summary, the minimum cutting thickness depends on the tool nose radius and the friction coefficient. Specifically, it exhibits a positive correlation with the tool nose radius and a negative correlation with the friction coefficient. By setting the tool nose radius within 0.01–0.03 mm and the friction coefficient within 0.1–0.6, the resulting influence curves depicting the minimum cutting thickness as a function of these two parameters are plotted in Figure 5.

## 3. Numerical Simulation Modeling

Metal cutting involves continuous material separation. Accurate simulation of chip formation and flow requires incorporating chip separation criteria into the finite element model. The influence of the tool nose radius is significant and cannot be neglected in micro-cutting due to the small scale involved. In simulation, this geometry disrupts the material flow directly ahead of the tool tip. The resulting intense plastic deformation can cause severe distortion in the mesh of the uncut workpiece region. Such mesh distortion not only compromises the accuracy of the results but can also create contact issues between the tool and workpiece, potentially leading to simulation non-convergence. To address this, the simulation utilizes the built-in Arbitrary Lagrangian-Eulerian (ALE) adaptive meshing method within Deform software (v11.0) to facilitate automatic chip separation. This method performs continuous automatic re-meshing, which prevents severe distortion of elements near the tool tip. Consequently, it enhances simulation convergence and improves computational efficiency.

### 3.1. Finite Element Model Setup

#### Workpiece and Tool Materials

The workpiece is made of 304 stainless steel bar stock. Its material properties are provided in Table 1, respectively. The cutting tool is fabricated from YG8 grade cemented carbide.

A material constitutive model defines its mechanical response by incorporating key deformation parameters such as strain, temperature, and strain rate. Consequently, the choice of constitutive model is critical for achieving accurate finite element simulation results. In metal cutting simulations, commonly used constitutive equations include the Zerilli-Armstrong model [30], the Bodner–Partom model [31], and the Johnson–Cook model [32]. The Johnson–Cook (J-C) model is particularly well-suited for this context as it accounts for the coupled effects of strain, strain rate, and temperature on the material’s flow stress. This makes it applicable for describing deformation under the large strains and high strain rates characteristic of machining processes. Therefore, the J-C constitutive model is adopted in this work. Its mathematical formulation is presented in Equation (12).

(12) $$\sigma =\left(A+B\varepsilon^{n}\right)\left[1+C\ln\left(\frac{\dot{\varepsilon }}{{\dot{\varepsilon }}_{0}}\right)\right]\left[1-{\left(\frac{T-T_{r}}{T_{m}-T_{r}}\right)}^{m}\right]$$

where ε is the equivalent plastic strain, $\dot{\varepsilon }$ is the equivalent plastic strain rate, ${\dot{\varepsilon }}_{0}$ is the reference strain rate, $T_{r}$ is the room temperature, $T_{m}$ is the melting temperature, T is the instantaneous deformation temperature, A is the initial yield stress, B is the strain hardening coefficient, C is the strain rate sensitivity coefficient, n is the work hardening exponent, and m is the thermal softening exponent. The specific values for these parameters in the J-C model are provided in Table 2.

The Johnson–Cook Damage failure model accurately simulates the failure and subsequent deletion of elements in the workpiece material during metal cutting simulations because it considers the effects of strain rate, temperature, and stress triaxiality on the fracture strain [34]. Consequently, the Johnson–Cook Damage model is selected as the material’s initial damage criterion. The damage evolution is defined by Equation (13):

(13) $$D=\sum \frac{\Delta \varepsilon^{pl}}{\varepsilon_{f}^{pl}}$$

where D is the damage variable, $\Delta \varepsilon^{pl}$ is the equivalent plastic strain increment, and $\varepsilon_{f}^{pl}$ is the equivalent plastic strain at failure. The expression for $\varepsilon_{f}^{pl}$ is provided by Equation (14) [35]:

(14) $$\varepsilon_{f}^{pl}=\left[D_{1}+D_{2}\exp\left(D_{3}\sigma^{*}\right)\right]\left[1+D_{4}l\overset{˙}{n}\varepsilon^{*}\right]\left[1+D_{5}\left(\frac{T-T_{r}}{T_{m}-T_{r}}\right)\right]$$

In this equation, $\sigma^{*}$ represents the stress triaxiality, p is the hydrostatic pressure, T is the instantaneous cutting temperature, $T_{r}$ is the room temperature (25 °C), $T_{m}$ is the melting temperature of the material, the reference strain rate is ${\dot{\varepsilon }}_{0}=1s^{-1}$, and D1 through D5 are the Johnson–Cook damage parameters.

The Coulomb friction model is implemented, defined by Equation (15):

(15) $$\tau_{f}=min\left(\mu \sigma_{n},\tau_{y}\right)$$

where $\tau_{f}$ is the frictional shear stress, $\sigma_{n}$ is the contact normal stress, $\tau_{y}$ is the material’s ultimate shear yield stress, and μ is the coefficient of friction. While the friction coefficient is typically determined experimentally, an empirical value of μ = 0.6 is used for this model [36].

**Figure 6:**
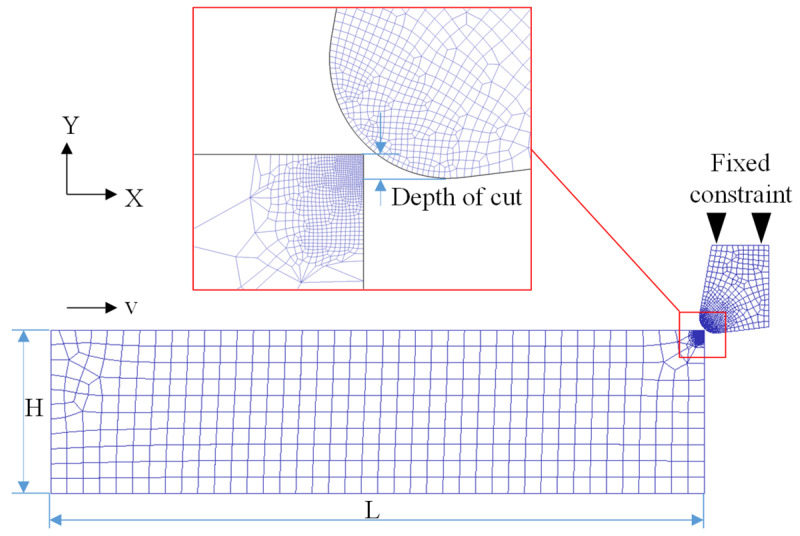
Two-dimensional orthogonal cutting finite element model.

The cutting tool is modeled as a rigid body featuring a 10° rake angle, a 7° clearance angle, and a tool nose radius of 20 μm. The workpiece has dimensions of 1.2 mm × 0.4 mm. A tetrahedral mesh is applied to the workpiece. To balance computational accuracy and efficiency, the mesh is refined in the vicinity of the tool tip. Based on initial simulation trials, the size of the refined elements is set to 2 μm. Assign the material parameters in Table 2 to the workpiece module. The finalized two-dimensional orthogonal cutting finite element model is illustrated in Figure 6.

### 3.2. Finite Element Model Validation

#### 3.2.1. Experimental Setup

**Figure 7:**
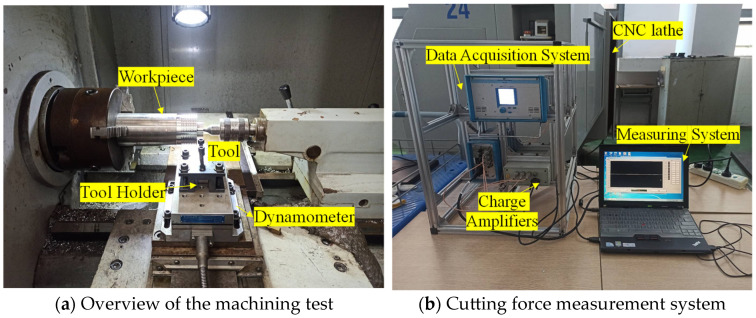
Photograph of the experimental setup.

Machining trials were performed on a C6136HK model CNC lathe. Cutting forces were measured using a Kistler 9257 B piezoelectric dynamometer (Kistler, Shanghai, China). A photograph of the experimental setup is presented in Figure 7.

#### 3.2.2. Experimental Design

The cutting parameters followed standard values recommended by the tool supplier for 304 stainless steel. The detailed experimental plan, along with the corresponding measured principal cutting forces, is summarized in Table 3.

#### 3.2.3. Simulation-Experiment Comparison and Validation

**Figure 8:**
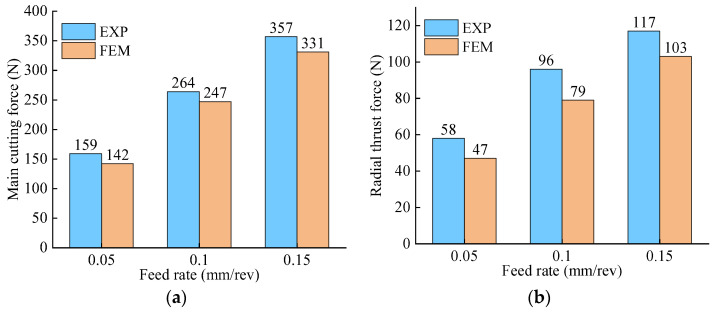
Cutting force comparison between simulation and experiment. (**a**) Main cutting force; (**b**) radial thrust force.

For validation purposes, experimental data from Groups 1, 2, and 3 of the test matrix were used. A comparison between the experimental measurements and the simulation results for the principal cutting force and the cutting resistance is presented in Figure 8. The simulated forces at various feed rates demonstrate a close match with the experimental data. The average error for the main cutting force in Figure 8a is calculated as 8.1%, and that for the thrust force in Figure 8b is 14.8%. These errors are attributable to the combined uncertainties in the material constitutive model, experimental conditions, simplified numerical modeling, and key input parameters. Nevertheless, the finite element results and experimental data show consistent trends. Given that errors below 15% are generally acceptable, the simulation accuracy of this model is considered reliable.

### 3.3. Effect of Depth of Cut on Material Separation

**Figure 9:**
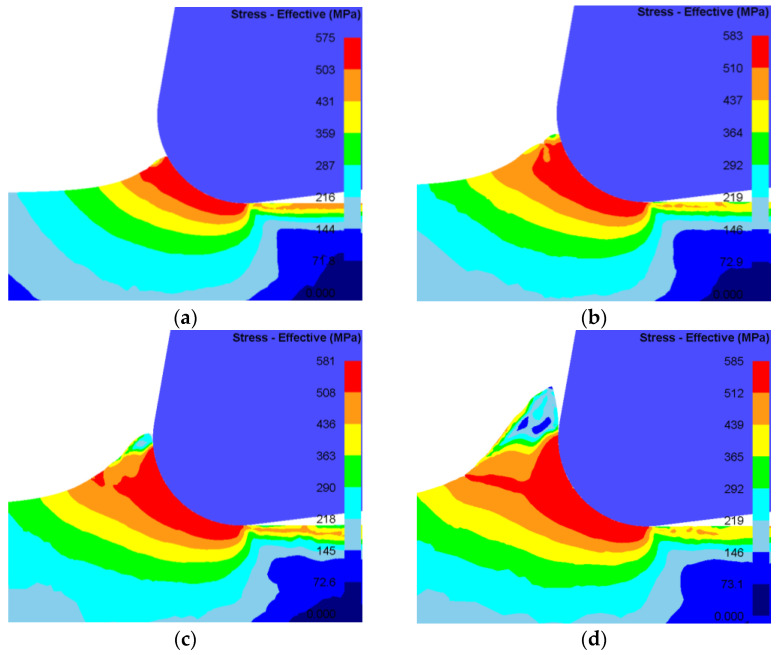
Contour plot of stress distribution under steady-state cutting conditions. (**a**) Depth = 2 μm; (**b**) depth = 3 μm; (**c**) depth = 4 μm; (**d**) depth = 5 μm.

Simulations were conducted with a cutting speed of 90 m/min and depths of cut corresponding to 0.1 r (2 μm), 0.15 r (3 μm), 0.2 r (4 μm), and 0.25 r (5 μm) of the tool nose radius, while keeping other parameters constant. The resulting simulated chip morphologies are presented in Figure 9. The analysis reveals that the maximum stress value on the generated machined surface remains essentially unchanged as the depth of cut increases. At a depth of 2 μm, deformation is primarily elastic. Material ahead of the tool is displaced via a pushing/extrusion mechanism without forming a continuous chip, and the induced stress field does not penetrate the uncut material. When the depth is increased to 4 μm, the stress begins to penetrate the uncut surface. In this regime, shearing starts to dominate over ploughing, leading to the initiation of chip formation. As the depth of cut increases further, chip formation becomes more stable, stress penetrates completely into the uncut workpiece, and a more pronounced stress band develops. Consequently, the penetration of the stress field into the uncut material serves as a key indicator for the onset of chip formation and material separation.

**Figure 10:**
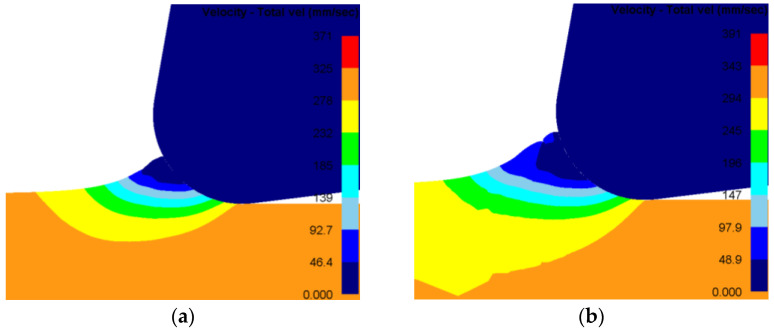

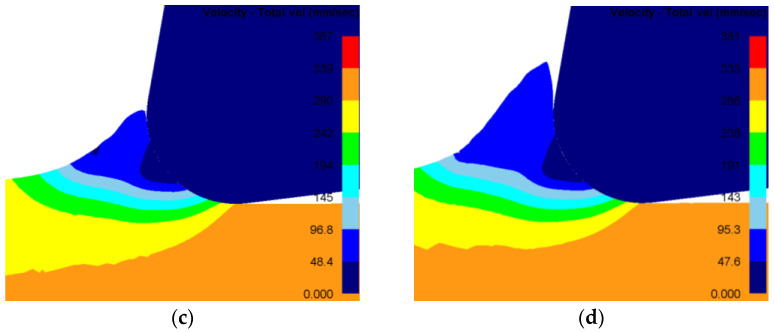
Contour plot of material flow velocity under steady-state cutting. (**a**) Depth = 2 μm; (**b**) depth = 3 μm; (**c**) depth = 4 μm; (**d**) depth = 5 μm.

Figure 10 presents the contour of material flow velocity ahead of the tool tip. A clear evolution in the flow pattern is observed as the depth of cut increases. For an actual depth of 2 μm, the material flows predominantly in the negative X-direction. At 4 μm, a distinct chip form emerges, with material flowing along the tool rake face in the positive X-direction. This confirms active chip formation, primarily governed by the interaction between the rake face and the workpiece. Notably, at depths of 4 μm and 5 μm, a triangular region of nearly stagnant material (flow velocity ≈ 0) develops ahead of the tool tip, identified as the DMZ. The height of this zone is often associated with the minimum cutting thickness for the given conditions. In these simulations, no stable DMZ forms at 2 μm, implying the depth is below the minimum cutting thickness. At 3 μm, the DMZ forms but is unstable throughout the cutting process. This instability suggests that the cutting thickness is very close to the material’s minimum cutting thickness, where the coupled effects of ploughing and shearing control deformation.

**Figure 11:**
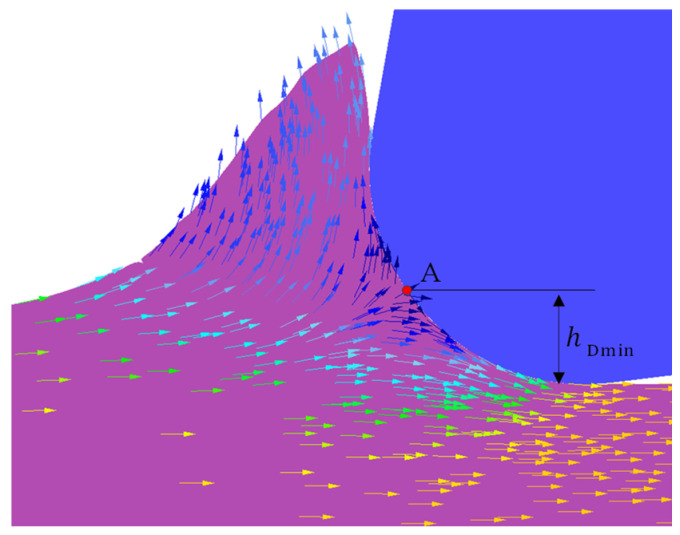
Velocity vector field plot (depth of cut = 5 μm, the arrow represents the vector direction of material flow, the color has no special meaning).

Analysis of the material flow velocity vector field in Figure 11 identifies a distinct point A at the boundary between the tool nose arc and the DMZ. Material above point A flows outwards along the tool rake face to form the chip, whereas material below this point is extruded downwards to constitute the machined surface. Point A is therefore defined as the material separation point. Measuring the vertical distance from this point to the machined surface yields a minimum cutting thickness of 5.5 μm. The ratio of this thickness to the tool nose radius is 0.275. Compared to the value of 4.6 μm predicted by the analytical model, the discrepancy is 16.4%. These results demonstrate that both the analytical model and the finite element model established in this work provide predictions of acceptable accuracy.

### 3.4. Effect of Cutting-Edge Micro-Geometry on Material Separation

To meet the demands of increasingly challenging machining scenarios, asymmetric cutting-edge profiles—like chamfered edges—are often generated through specialized edge preparation or passivation processes. These micro-geometric features, designed to enhance edge strength and reduce vibrations, also significantly impact the material separation process. The alteration in separation behavior subsequently modifies the distributions of stress and temperature in the cutting zone, thereby affecting overall tool performance. Based on the finite element model developed previously, this section presents a comparative analysis of how variations in the tool nose radius and edge morphology influence material separation.

#### 3.4.1. Influence of Tool Nose Radius on Material Separation

**Figure 12:**
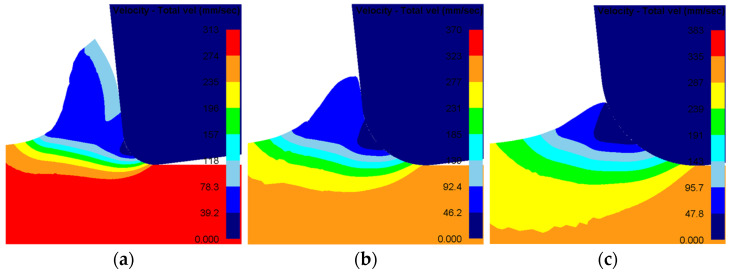
Material flow distribution for tools with different tool nose radius. (**a**) r = 0.01 mm; (**b**) r = 0.02 mm; (**c**) r = 0.03 mm.

Simulations were configured with a tool rake angle of −6°, a clearance angle of 7°, and a constant depth of cut of 5 μm. Three tool nose radii were investigated: 0.01 mm, 0.02 mm, and 0.03 mm. All other finite element model parameters matched those defined in Section Workpiece and Tool Materials. Figure 12 presents the extracted material flow velocity contours for these three cases. Analysis shows that at the 5 μm depth of cut, chips are generated for both the 0.01 mm and 0.02 mm nose radii, confirming that the cutting depth meets or exceeds the minimum cutting thickness. The chip formation is especially pronounced for the 0.01 mm radius, exhibiting precise flow along the rake face and a well-defined stress band that penetrates the machined surface ahead of the tool. Conversely, with the 0.03 mm nose radius, no such penetrating stress band is observed. Material separation occurs mainly via a pushing/extrusion process; material above the separation point flows along the nose arc, with minimal flow directed up the rake face. This behavior indicates that the 5 μm depth of cut is approximately equal to the minimum cutting thickness for the tool with the 0.03 mm nose radius.

**Figure 13:**
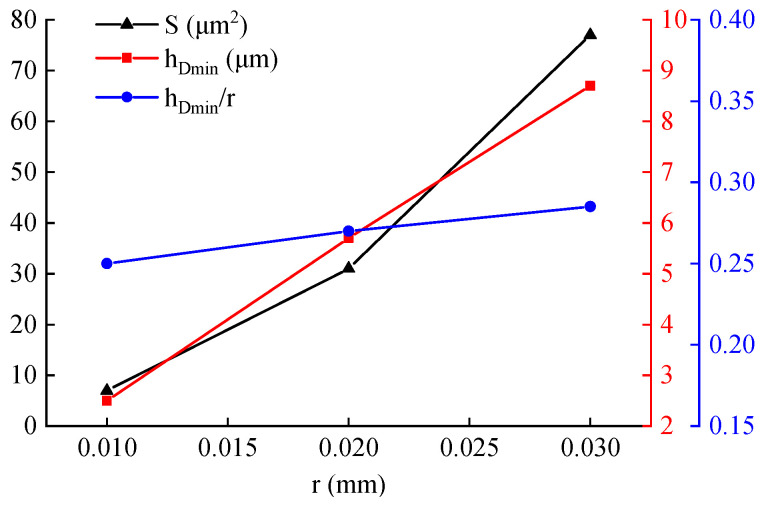
Comparison of key simulation outputs for different tool nose radius.

A comparison of the DMZ characteristics under different tool nose radii reveals a positive correlation between the DMZ size and the radius. Specifically, a larger tool nose radius results in a more extensive DMZ during cutting. For each radius, key parameters were quantified: the DMZ area, the distance from the material separation point to the machined surface, and the ratio of the minimum cutting thickness to the tool nose radius. These results are compiled in Figure 13. The data show that both the minimum cutting thickness and the DMZ area increase with a larger tool nose radius. This trend implies an enhanced ploughing effect as the radius grows, leading to greater accumulation of workpiece material immediately ahead of the edge. In contrast, the ratio of the minimum cutting thickness to the tool nose radius remains relatively stable across the tested conditions, fluctuating within a narrow range of 0.25 to 0.30.

#### 3.4.2. Edge Morphology Characterization Method

**Figure 14:**
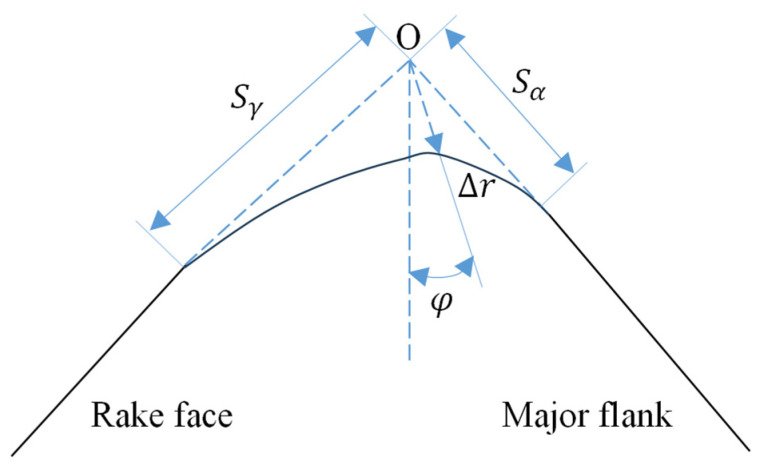
Schematic of the K-factor characterization method.

The profile of a cutting-edge resulting from preparation and passivation processes is rarely a simple arc; it frequently consists of multiple interconnected complex curves. The intricacy of edge measurement and characterization often necessitates several parameters for an accurate description, and a universally accepted standard method is lacking. A frequently used yet overly simplistic approach approximates the rake-flank transition zone with an ideal circle, which can introduce substantial error. Wyen introduced an alternative method for describing the edge geometry [37], which allows for the unambiguous definition of a honed edge’s dimensions and was used to analyze cutting forces and friction coefficients for tools with different hone radii. For characterizing complex edge profiles, Denkena [13] stated that this technique characterizes the edge using four parameters. The rake and flank face contours are extended until they intersect at a point considered the virtual sharp tip. The distances from this tip to the start points of the extended rake and flank contours are denoted $S_{\gamma }$ and $S_{\alpha }$, respectively. The minimum distance from the tip to the actual edge profile is ∆r, and φ is the angle between the extension lines and the bisector of the tip wedge angle. The K-factor is defined as the ratio K = $S_{\gamma }/S_{\alpha }$. An edge is symmetric when K = 1. A K value less than 1 describes a waterfall-type edge, while a value greater than 1 indicates a trumpet-type edge. The schematic of the K-factor characterization method as shown in Figure 14.

#### 3.4.3. Effect of Asymmetric Edge Morphology on Material Separation

**Figure 15:**
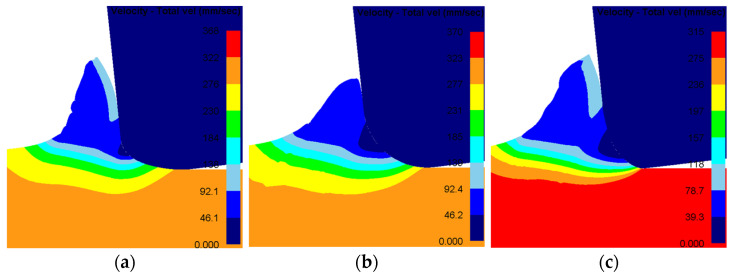
Material flow distribution for tools with different K-factors. (**a**) K = 0.5; (**b**) K = 1.0; (**c**) K = 0.15.

**Figure 16:**
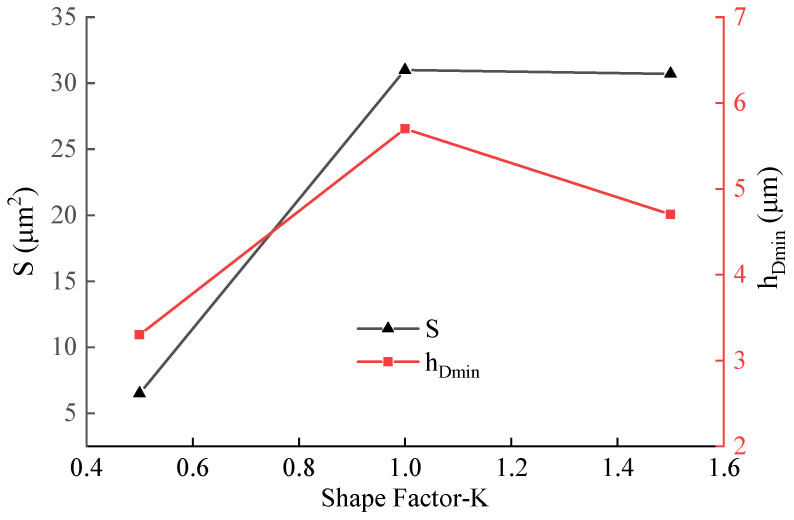
Comparison of DMZ area and minimum cutting thickness for different K-factors.

Three tool geometries were created with edge shape factor (K-factor) values of 0.5, 1.0, and 1.5, and simulated using the finite element software. The material flow velocity fields ahead of the tool tip for each K-factor were obtained, and the DMZ area and minimum cutting thickness were measured. These results are presented in Figure 15 and Figure 16, respectively. The analysis reveals that variations in the edge micro-geometry substantially alter the material deformation and flow patterns near the tool tip. For K < 1, both the DMZ area and the minimum cutting thickness increase with the K-factor. When K > 1, the DMZ area shows no significant correlation with K, remaining relatively constant; in contrast, the minimum cutting thickness tends to decrease as K increases. Generally, a higher K-factor produces an edge profile closer to a negative chamfer. This geometry induces greater chip deformation and causes a more abrupt change in the material flow direction within the DMZ.

## 4. Simulation Analysis of Microstructure Evolution

### 4.1. Development of the Dislocation Density-Based Model

Finite element modeling for predicting the evolution of microstructure in machined surfaces primarily involves two approaches. One type aims to predict the work-hardened layer beneath the machined surface [38], focusing on how the altered subsurface microstructure affects properties such as microhardness. The other type of model’s microstructural transformation is under severe plastic deformation [39], predicting changes in grain size within both the chip and the machined surface layer. The dislocation density theory, introduced by Estrin et al., effectively describes material hardening under large-strain conditions. Leveraging this theory, the present work employs the user subroutine interface in ABAQUS to develop and implement a custom subroutine. This dislocation density-based subroutine is integrated into the 2D orthogonal turning finite element model to simulate and predict the dislocation density, grain size, and microhardness in the machined surface.

The predictive model, based on dislocation density theory, quantifies the extent of plastic deformation directly in terms of dislocation density. The dislocation cell structure that develops during plastic deformation comprises two distinct densities: the density within the cell interiors ($\rho_{c}$) and the density in the cell walls ($\rho_{w}$). The wall dislocation density is further subdivided into statistically stored dislocations ($\rho_{ws}$) and geometrically necessary dislocations ($\rho_{wg}$). Their governing equations are presented below:

(16) $${\dot{\rho }}_{c}=\alpha^{*}\left(\frac{1}{\sqrt{3}b}\right)\sqrt{\left(\rho_{ws}+\rho_{wg}\right)}{\dot{\gamma }}_{w}^{r}-6\beta^{*}{\left(dbf{\left(1-f\right)}^{\frac{1}{3}}\right)}^{-1}{\dot{\gamma }}_{c}^{r}-k_{0}{\left(\frac{{\dot{\gamma }}_{w}^{r}}{{\dot{\gamma }}_{0}}\right)}^{-\frac{1}{n}}\rho_{c}{\dot{\gamma }}_{c}^{r}$$

(17) $${\dot{\rho }}_{w}=\beta^{*}\frac{\sqrt{3}\left(1-f\right)}{bf}\sqrt{\left(\rho_{ws}+\rho_{wg}\right)}{\dot{\gamma }}_{c}^{r}+\beta^{*}\frac{6\left(1-f\right)\frac{2}{3}}{dbf}{\dot{\gamma }}_{c}^{r}-k_{0}{\left(\frac{{\dot{\gamma }}_{c}^{r}}{{\dot{\gamma }}_{0}}\right)}^{-\frac{1}{n}}\rho_{ws}{\dot{\gamma }}_{w}^{r}$$

In the equations, ξ is the fraction of geometrically necessary dislocations within the cell walls; $\alpha^{*}$, $\beta^{*}$, and $k_{0}$ are material parameters governing dislocation evolution rates, Their values are 0.0946, 0.0113 and 8.462 [40]; the initial dislocation densities are set to $\rho_{w0}$ = 10^7^ mm^−2^ for the cell walls and $\rho_{c0}$ = 10^8^ mm^−2^ for the cell interiors [41], ${\dot{\gamma }}_{0}$ is a reference shear strain rate; b is the Burgers vector magnitude; and n is a temperature sensitivity parameter defined in Equation (18):

(18) $$n=\frac{T}{B}$$

f denotes the volume fraction of cell wall dislocations, as given by Equation (19):

(19) $$f=f_{\infty }+\left(f_{0}-f_{\infty }\right)\exp\left(-\frac{\gamma^{r}}{{\bar{\gamma }}^{r}}\right)$$

where $f_{0}$ and $f_{\infty }$ represent the initial and saturated volume fractions of the dislocation cell walls, respectively; $\gamma^{r}$ is the shear strain; and ${\bar{\gamma }}^{r}$ is the reference plastic strain. It is generally assumed that the shear strain rates within the material are equal, as expressed in Equation (20):

(20) $${\dot{\gamma }}_{w}^{r}={\dot{\gamma }}_{c}^{r}={\dot{\gamma }}^{r}$$

where ${\dot{\gamma }}_{w}^{r}$ is the shear strain rate in the dislocation cell walls, and ${\dot{\gamma }}_{c}^{r}$ is the shear strain rate within the cell interiors. The shear strain rate is given by Equation (21):

(21) $${\dot{\gamma }}^{r}=M\dot{\varepsilon }$$

where M is the Taylor factor, and $\dot{\varepsilon }$ is the plastic strain rate. The total dislocation density $\rho_{tot}$ is determined from the dislocation cell densities and their volume fractions, as shown in Equation (22):

(22) $$\rho_{tot}=f\left(\rho_{ws}+\rho_{wg}\right)+\left(1+f\right)\rho_{c}$$

The grain size d is described by Equation (23), where K is a material constant.

(23) $$d=\frac{K}{\sqrt{\rho_{tot}}}$$

Estrin et al. enhanced the dislocation density-based model for material flow stress originally proposed by Kocks [42]. Their refinement involves decomposing the overall shear stress into contributions from dislocations in the cell interiors and those in the cell walls, as detailed in Equations (24)–(26):

(24) $$\tau_{c}^{r}=Gab\sqrt{\rho_{c}}{\left(\frac{{\dot{\gamma }}_{c}^{r}}{{\dot{\gamma }}_{0}}\right)}^{\frac{1}{m}}$$

(25) $$\tau_{w}^{r}=Gab\sqrt{\rho_{w}}{\left(\frac{{\dot{\gamma }}_{w}^{r}}{{\dot{\gamma }}_{0}}\right)}^{\frac{1}{m}}$$

(26) $$\tau^{r}=f\tau_{w}^{r}+\left(1-f\right)\tau_{c}^{r}$$

G is the shear modulus, a is a material constant, and m is the temperature-dependent strain rate sensitivity parameter defined by Equation (27), where A is a constant.

(27) $$m=\frac{A}{T}$$

Severe plastic deformation characterizes the machined surface layer in metal cutting. The resulting microhardness profile is a consequence of the interplay between this deformation, potential phase transformations, and microstructural evolution. Thus, investigating microhardness must take into account microstructural changes. For dry cutting of AISI 304 stainless steel, the evolution of microhardness in the machined surface can be predicted by the dislocation density model through the following relation [43]:

(28) $$HV=k_{h}\times M\times \alpha_{h}\times G\times b\times \sqrt{\rho_{tot}}$$

$k_{h}$ is a slope constant equal to 0.5, and $\alpha_{h}$ is a constant with a value of 0.25 [44].

### 4.2. Validation of the Microstructure Evolution Model

**Figure 17:**
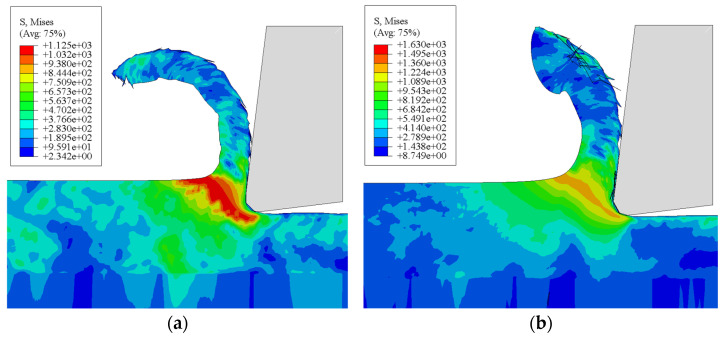
Comparison of von Mises stress distributions. (**a**) The dislocation density model; (**b**) the J-C model (Gray represents the tool and the black line represents the mesh distortion section).

**Figure 18:**
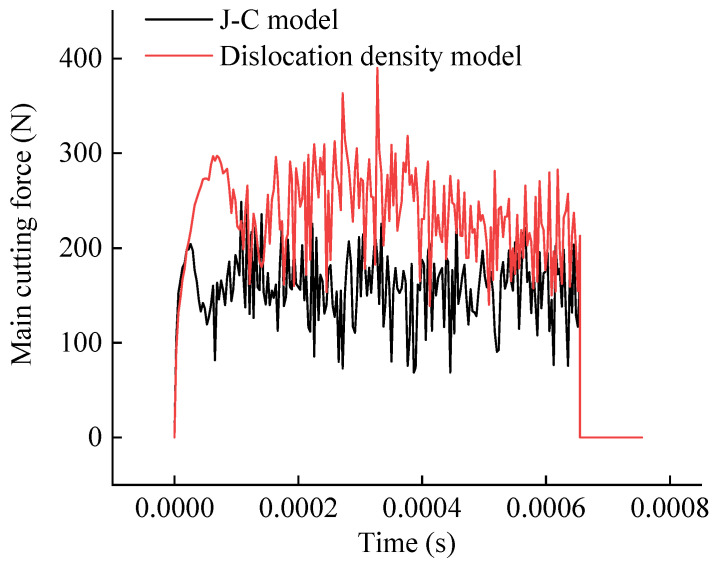
Comparison of the predicted principal cutting force and von Mises stress history.

The parameters for the dislocation density model were initialized. A user material subroutine (Vuhard) implementing this model was developed and integrated into the established 2D orthogonal cutting finite element framework. Its predictions were then compared against those from the built-in J-C constitutive model in Abaqus, focusing on principal cutting force and von Mises stress. Figure 17 displays contour plots of von Mises stress from both models for a specific cutting condition (v = 150 m/min, f = 0.1 mm/rev, ap = 1 mm). The comparison focuses on identical material points within the primary shear zone at a simulation time step corresponding to the mid-point of the cutting process. The temporal evolution of the principal cutting force and the von Mises stress for both models is plotted in Figure 18.

The results indicate that both models predict very similar trends for the principal cutting force over time. However, at comparable time steps, the dislocation density model predicts a more intense stress concentration within the primary shear zone than the J-C model. Notably, the cutting force predicted by the dislocation density model aligns more closely with experimentally measured values.

### 4.3. Morphological Analysis of the Machined Subsurface Microstructure

**Figure 19:**
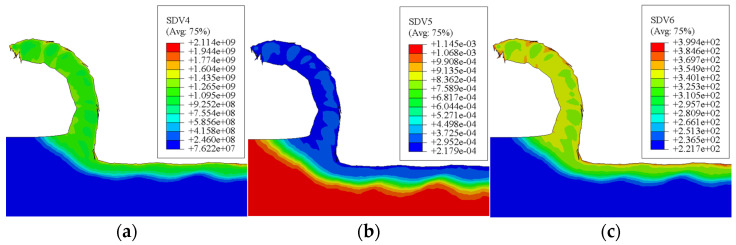
Predicted microstructure evolution in the machined subsurface. (**a**) Dislocation density distribution; (**b**) grain size distribution; (**c**) microhardness distribution.

Machining induces the formation of a modified surface layer on metallic workpieces. An elevated dislocation density and refined grain structure characterize this altered layer. Figure 19 illustrates simulated contour maps of this subsurface region for a specific cutting condition (v = 150 m/min, f = 0.10 mm/rev, ap = 1 mm). The plots correspond to user-defined output variables: SDV4 for dislocation density (mm^−2^), SDV5 for grain size (mm), and SDV6 for microhardness (HV). The results confirm significant grain refinement at the immediate surface. With increasing depth below the machined surface, the dislocation density and microhardness decrease progressively, while the grain size increases, eventually reaching the properties of the unaffected bulk material.

**Figure 20:**
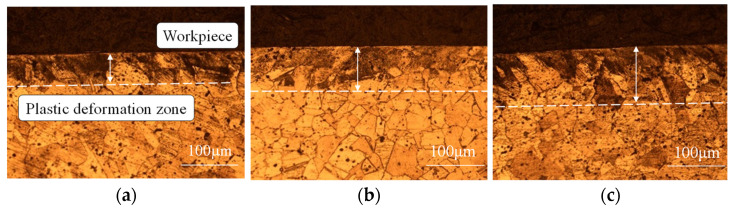
Microstructure of surface layers at different cutting speeds. (**a**) v = 90 m/min; (**b**) v = 150 m/min; (**c**) v = 210 m/min.

Under intense thermo-mechanical coupling, the subsurface microstructure of the machined surface transforms. Figure 20 presents the microstructural characteristics of the surface layer at different cutting speeds. To delineate the plastic deformation, white dashed lines mark the boundary between the slip bands and the matrix material, defining the plastic deformation zone. The figure shows that grain deformation in the surface layer increases significantly. Numerous slip lines aggregate to form slip bands, and the density of these slip lines gradually decreases with depth, exhibiting a non-uniform distribution.

Severe work hardening occurs during the machining of 304 stainless steel. In this material, plastic deformation within the microstructure of the machined surface primarily manifests as slip bands [45]. The fundamental mechanism of work hardening in metals involves an alteration of the crystalline microstructure, specifically an increase in resistance to dislocation motion with mechanical deformation. Consequently, the extent of work hardening is directly linked to the evolution of the microstructure. Following the cutting experiments, a 3 mm sample section was obtained from the workpiece using wire electrical discharge machining. This sample was vertically mounted in an XB-2B model metallographic mounting press. After being ground to a flat and smooth finish, the specimen was subjected to microhardness testing. A DHV-1000Z digital microhardness tester (Shanghai Precision Instrumentation Company, Shanghai, China) was used to measure at 20 µm intervals along the depth from the machined surface. Three measurements were taken at each depth, and the average value was recorded as the experimental result. The scatter among repeated measurements was quantified using the standard deviation, which reflects the combined effects of local microstructural heterogeneity, indentation size effects, and surface preparation quality. This variability is unavoidable in microhardness testing of heavily deformed surface layers and is therefore explicitly accounted for in the interpretation of the results.

**Figure 21:**
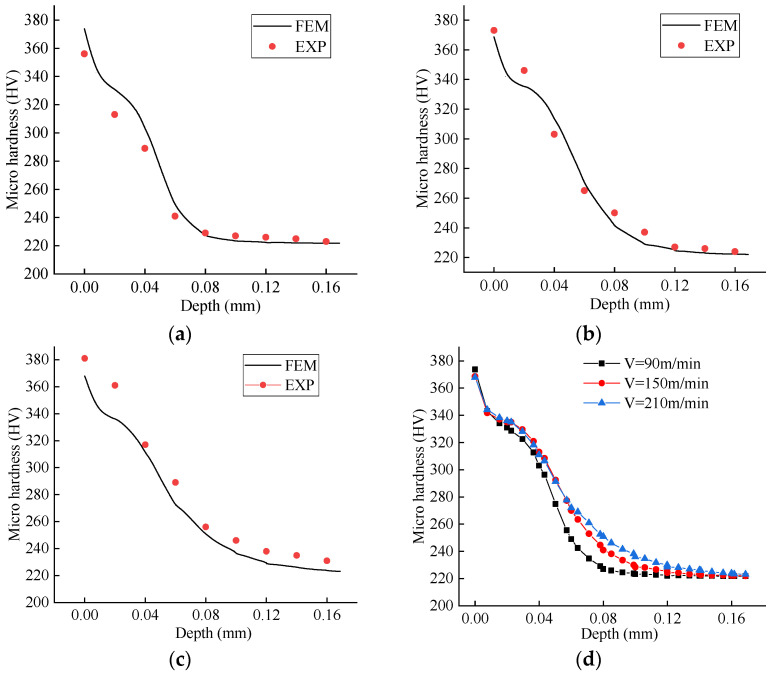
Comparison of microhardness. (**a**) v = 90 m/min, f = 0.1 mm/rev; (**b**) v = 150 m/min, f = 0.1 mm/rev; (**c**) v = 210 m/min, f = 0.1 mm/rev; (**d**) machined surface hardness vs. depth at different cutting speeds.

Figure 21a–c compare the experimental and simulated microhardness profiles along the depth beneath the machined surface for different cutting speeds. The results show that the microhardness gradually decreases with increasing depth, with the work-hardening effect diminishing to approximately the base-material hardness at depths of around 100–120 µm. The extracted microhardness variation trends for different cutting speeds are shown in Figure 21d. At a given depth, the microhardness increases with higher cutting speeds, especially when the depth is between 40 and 120 μm; however, the incremental increase becomes progressively smaller. This observation corroborates the idea that work hardening results from the combined influence of two competing factors: the strengthening effect of plastic deformation and the softening effect induced by cutting heat. This behavior is related to the ratio of the material’s hardening rate to its softening rate during the cutting process. It is noted that some references report an opposite trend, in which the machined surface microhardness decreases with increasing cutting speed [46]. In summary, the microhardness prediction model developed in this work demonstrates good agreement with experimental data and performs reliably. It should be noted that the comparison focuses primarily on the consistency of the overall depth-dependent trends rather than exact point-by-point agreement, given the inherent experimental scatter in microhardness measurements near the machined surface.

### 4.4. Impact of Cutting-Edge Micro-Geometry on Microstructure Evolution

#### 4.4.1. Effect of Tool Rake Angle on Machined Surface Microstructure

**Figure 22:**
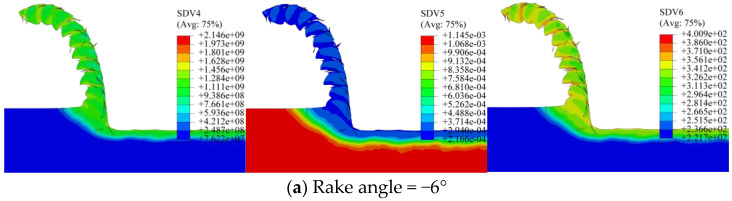

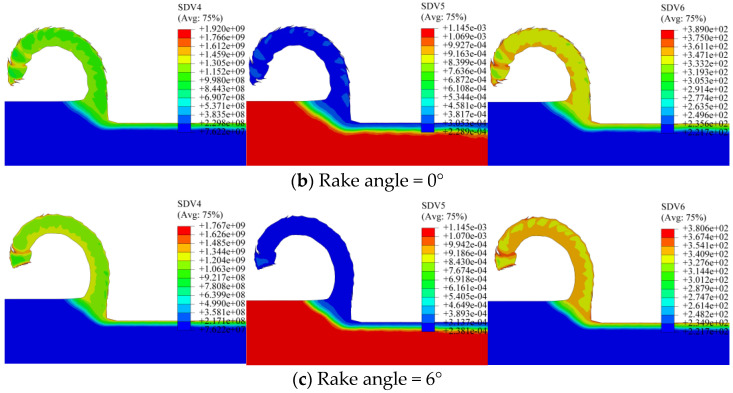
Simulated subsurface distributions for different tool rake angles. (**a**) Rake angle = −6°; (**b**) rake angle = 0°; (**c**) rake angle = 6°.

A quantitative analysis was conducted to evaluate the impact of the rake angle on dislocation density, grain size, and microhardness in the machined layer. Finite element simulations were run with rake angles of −6°, 0°, and 6°, keeping all other parameters identical. The previously developed dislocation density subroutine was integrated into the 2D orthogonal turning model. Figure 22 displays the simulated distributions of these three microstructural parameters for each rake angle. The results indicate that the peak dislocation density is localized near the tool-workpiece contact point at the surface. All three properties—dislocation density, grain size, and microhardness—vary with depth from the machined surface, eventually approaching the values of the bulk material. The measured extremes for each case are as follows: for rake angles of −6°, 0°, and 6°, the maximum dislocation densities are 2.146 × 10^9^ mm^−2^, 1.920 × 10^9^ mm^−2^, and 1.767 × 10^9^ mm^−2^; the minimum grain sizes are 0.2166 μm, 0.2289 μm, and 0.2381 μm; and the maximum microhardness values are 400.9 HV, 389.0 HV, and 380.6 HV, respectively.

**Figure 23:**
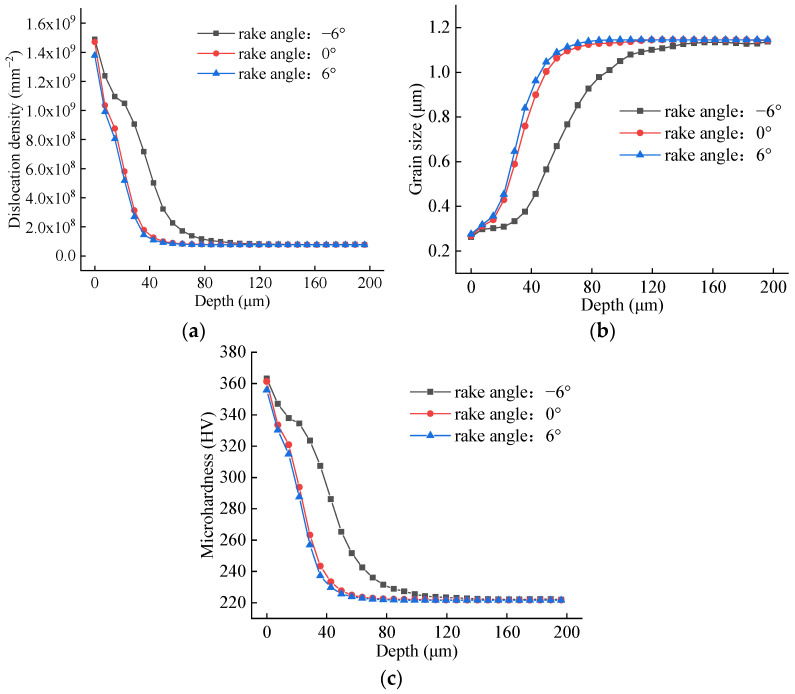
Averaged depth profiles for different tool rake angles. (**a**) Dislocation depth profile; (**b**) grain size depth profile; (**c**) microhardness depth profile.

To quantify the subsurface gradients, data were extracted along three 200 μm long line paths defined normal to the machined surface. The nodal values of dislocation density, grain size, and microhardness along each path were averaged to obtain representative depth profiles. Figure 23 presents these averaged simulation results, illustrating the effect of the tool rake angle on the distributions under constant cutting conditions. The profiles indicate that increasing the rake angle results in a systematic decrease in dislocation density and microhardness, accompanied by an increase in grain size, across the affected depth. The gradients are markedly steeper for negative rake angles (in the range [−6°, 0°]) compared to positive ones ([0°, 6°]).

Additionally, the penetration depth of the altered layer diminishes as the rake angle increases. The calculated average altered layer depths are 133 μm, 77 μm, and 56 μm for rake angles of −6°, 0°, and 6°, respectively. These results confirm that a more minor, especially negative, rake angle promotes more intense plastic deformation during cutting, which in turn is more effective in refining the grain structure.

#### 4.4.2. Effect of Tool Nose Radius on Machined Surface Microstructure Evolution

**Figure 24:**
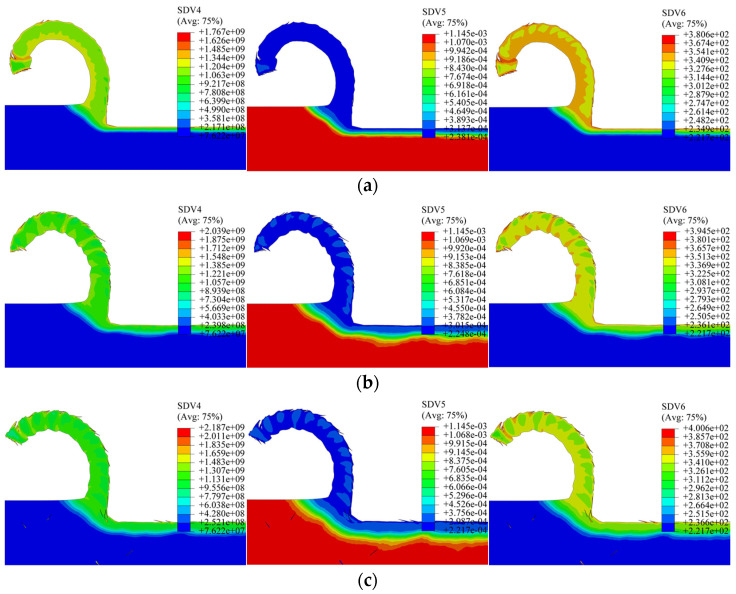
Contour plots for different tool nose radius. (**a**) r = 0.02 mm; (**b**) r = 0.03 mm; (**c**) r = 0.04 mm.

Simulations were performed with tool nose radii of 0.02 mm, 0.03 mm, and 0.04 mm, with all other cutting parameters held constant. Figure 24 presents the simulated contour maps of dislocation density, grain size, and microhardness for these three cases. The key simulation outputs are for radii of 0.02, 0.03, and 0.04 mm; the peak subsurface dislocation densities are 1.767 × 10^9^, 2.039 × 10^9^, and 2.187 × 10^9^ mm^−2^; the corresponding minimum grain sizes are 0.2381, 0.2248, and 0.2217 μm; and the maximum microhardness values are 380.6, 394.5, and 400.6 HV. A visual assessment also suggests that the depth of the altered layer increases with a larger nose radius.

**Figure 25:**
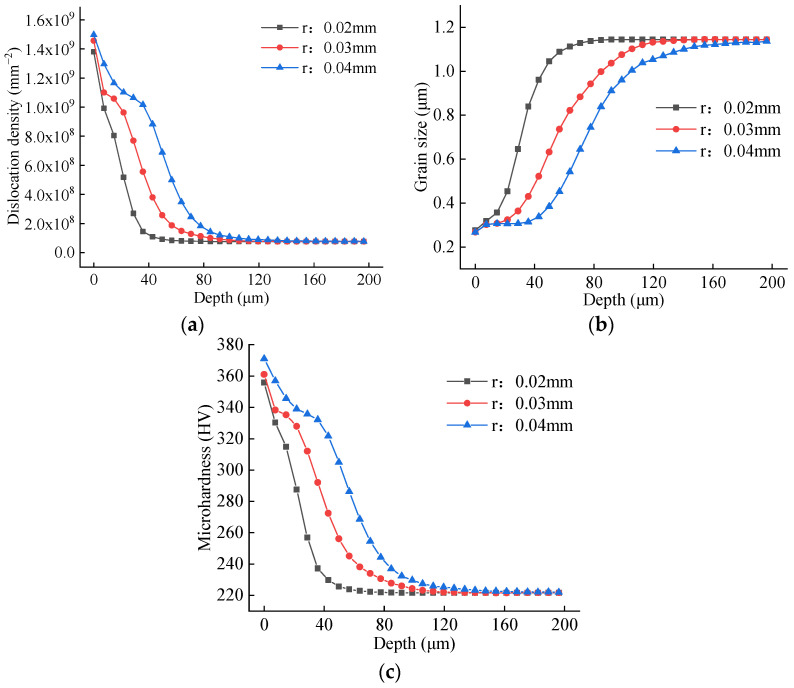
Averaged depth profiles for different tool nose radius. (**a**) Dislocation depth profile; (**b**) grain size depth profile; (**c**) microhardness depth profile.

Following the same methodology, averaged depth profiles were obtained along three 200 μm paths normal to the machined surface. These profiles, which illustrate the effect of nose radius under constant cutting conditions, are plotted in Figure 25. The data clearly show that a larger tool nose radius results in higher dislocation density and microhardness, along with smaller grain size, throughout the affected depth. Quantitatively, the average penetration depths of the altered layers are measured as 56 μm, 112 μm, and 147 μm for nose radii of 0.02 mm, 0.03 mm, and 0.04 mm, respectively. These findings demonstrate that a larger tool nose radius exacerbates plastic deformation during the cutting process. This intensified deformation promotes greater grain refinement, leading to increased dislocation density and microhardness in the machined surface layer.

#### 4.4.3. Effect of Asymmetric Edge Morphology on Machined Surface Microstructure Evolution

**Figure 26:**
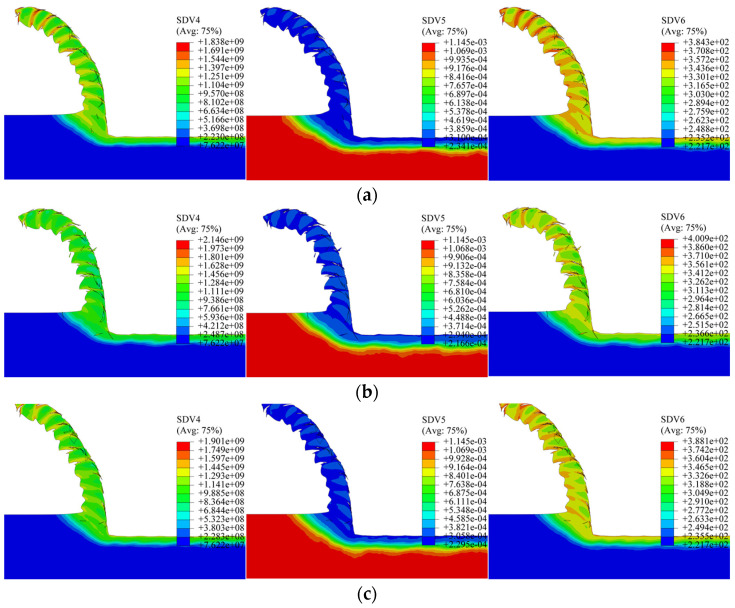
Contour plots for different K-factors. (**a**) K = 0.5; (**b**) K = 1; (**c**) K = 1.5.

Simulations were conducted with the edge shape factor (K-factor) set to 0.5, 1.0, and 1.5, maintaining identical cutting parameters otherwise. Figure 26 displays the corresponding simulated distributions of dislocation density, grain size, and microhardness. The key metrics for K = 0.5, 1.0, and 1.5 are: maximum dislocation densities of 1.838 × 10^9^, 2.146 × 10^9^, and 1.901 × 10^9^ mm^−2^; minimum grain sizes of 0.2341, 0.2166, and 0.2295 μm; and peak microhardness values of 384.3, 400.9, and 388.1 HV.

**Figure 27:**
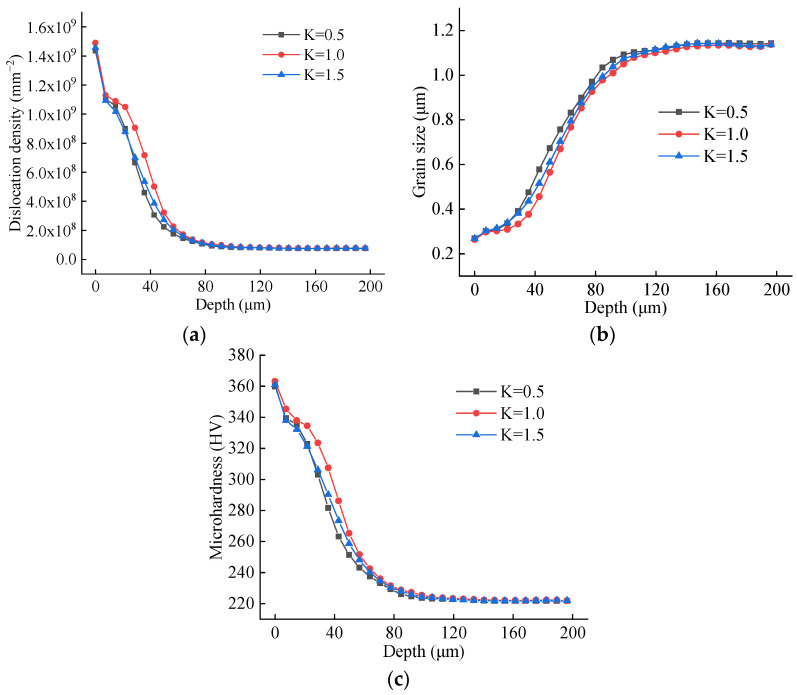
Averaged depth profiles for different K-factors. (**a**) Dislocation depth profile; (**b**) grain size depth profile; (**c**) microhardness depth profile.

Averaged depth profiles along three standard 200 μm paths were generated to analyze the influence of the K-factor, as plotted in Figure 27. The profiles exhibit a non-monotonic relationship: with increasing K-factor, both dislocation density and microhardness initially rise and then decline, while grain size initially decreases and then increases. The depth of the altered layer shows a relatively weak dependence on K, with average values of 119 μm, 133 μm, and 126 μm for K = 0.5, 1.0, and 1.5, respectively—also following an initial increase followed by a decrease. This pattern aligns with the previously observed trend of the minimum cutting thickness versus K-factor. The results suggest that a symmetric edge (K = 1) promotes more intense plastic deformation and consequently greater grain refinement compared to asymmetric edges (K ≠ 1). Comparing the magnitudes of change across all studied parameters, the tool nose radius and the rake angle are identified as the dominant factors governing subsurface microstructure evolution, with the nose radius exhibiting a more decisive influence than the rake angle.

#### 4.4.4. Recommendations for Cutting Process Optimization and Applicability Analysis

The results presented above indicate that the variations in dislocation density, grain size, and microhardness on the machined surface during the turning of 304 stainless steel are fundamentally governed by material separation behavior. The micro-geometry of the cutting edge further affects the extent of work hardening by modifying the separation point location and the proportion of ploughing action. Consequently, based on the patterns established in the previous sections, this section puts forward optimization recommendations for cutting processes and tool structures, with a focus on engineering applications. It also delineates the applicability scope and potential limitations of the derived conclusions, thereby enhancing the transferability and practical guidance value of the research findings.

(1) Recommendations for Cutting-Edge Micro-geometry Optimization Targeting Surface Integrity

I. The preceding results demonstrate that the tool nose radius exerts the most pronounced influence on microstructural evolution. In machining 304 stainless steel, an overly small edge radius, while beneficial for reducing ploughing, often compromises edge strength and induces fluctuations in surface loading. Conversely, a huge edge radius amplifies the adverse rake angle effect, forcing material removal to rely more heavily on extrusion and ploughing mechanisms. This results in increased plastic strain accumulation within the surface layer and a deeper work-hardened zone. Therefore, provided that adequate edge strength and stability are maintained, a medium edge radius should be prioritized to sustain a relatively stable material separation mode during cutting.

II. By modifying the effective rake angle distribution and the load transfer mode in the contact region, an asymmetric cutting edge significantly alters the separation point migration and the morphology of the local deformation zone, consequently leading to variations in the minimum uncut chip thickness and the extent of surface hardening. For engineering applications aiming to reduce surface damage and stabilize microstructural evolution, tool configurations that cause pronounced material accumulation and stagnation due to edge asymmetry should be avoided. Instead, appropriate K-factor matching can optimize the material separation path for smoother flow, mitigating additional loads from repeated extrusion and elastoplastic deformation. Thus, a nearly symmetric edge profile is recommended where tool strength permits. When a strongly asymmetric structure is necessary to enhance edge strength, cutting parameters must be co-optimized to offset its tendency to cause excessive surface hardening.

III. For work-hardenable materials like 304 stainless steel, surface layer hardening presents a dual character: moderate strengthening can improve wear and fatigue resistance, whereas excessive strengthening raises the risk of residual tensile stresses and undermines service reliability. Consequently, during edge parameter optimization, evaluation criteria should include metrics such as the peak dislocation density, the hardened layer depth, and the spatial extent of grain refinement. This shifts the paradigm for tool micro-geometry design from the conventional minimization of cutting forces or temperatures towards the targeted regulation of surface integrity.

(2) Recommendations for Process Parameter Optimization Aiming at Stable Cutting

I. The instantaneous undeformed chip thickness should be maintained above the range of the minimum uncut chip thickness. Operating near this minimum value makes cutting forces more susceptible to fluctuation, leading to significant instability in dislocation density and microhardness distributions. Therefore, the feed rate and tool edge parameters should be co-optimized to keep the cutting process within the stable regime of continuous chip formation, thus minimizing the risks of non-uniform surface layer hardening and surface damage.

II. Increasing the cutting speed helps reduce material stagnation and adhesion tendencies, but the accompanying thermal softening effect must be balanced. While the high thermal load at elevated speeds aids chip formation, it can also induce austenite-to-martensite phase transformation and enhance thermal softening. Hence, for applications demanding strict phase transformation control and the preservation of work hardening, a medium cutting speed (80–120 m/min) is recommended to achieve controlled grain refinement primarily through mechanical loading.

III. Although the present model does not incorporate fluid media, the predicted distribution of high-temperature zones—concentrated mainly ahead of the cutting edge and in the primary deformation zone—suggests that efficient cooling and lubrication methods, such as high-pressure coolant or minimum quantity lubrication (MQL), are beneficial. Their key purpose extends beyond mere temperature reduction; more critically, they modify the friction conditions at the tool-chip interface. This directly affects the shear angle and deformation zone morphology identified in the simulations, helping to mitigate surface integrity uncertainties arising from friction fluctuations and thereby enhancing the transferability of the model’s conclusions to practical manufacturing conditions.

(3) Scope of Applicability and Limitations

Although this work has developed a relatively comprehensive framework—integrating analytical modeling, finite element simulation, and experimental validation—for predicting material separation and microstructural evolution, its applicability is subject to certain boundaries, primarily in the following areas:

I. Material and Constitutive Parameter Dependency: The thermo-mechanical response and work hardening behavior of 304 stainless steel are sensitive to material batch and heat treatment history. Variations in the initial microstructure, strength level, or hardening characteristics can lead to deviations in the predicted absolute values of dislocation density and hardness. However, the general trends and patterns identified retain their value as references.

II. Simplification of Friction and Interface Conditions: In actual cutting, tool-chip contact friction is dynamic, changing with temperature, pressure, and the formation of adhesive layers. The finite element model employs simplified friction assumptions; consequently, its predictive accuracy may decrease under severe wear conditions.

III. Incomplete Consideration of Cutting-Edge Micro-defects and Wear Evolution: Real cutting edges can exhibit micro-chipping, coating delamination, or wear-induced deviations from the nominal edge geometry. These factors alter the material-separation mode and the subsequent microstructural evolution in the surface layer. Therefore, the conclusions drawn here are most applicable to machining stages characterized by stable edge conditions and controlled wear.

IV. Limits of Generalizability: This study focuses on typical operating parameters and a specific range of cutting-edge micro-geometries for turning 304 stainless steel. Extending the framework to significantly different conditions—such as minimal depths of cut, ultra-high speeds, or alternative tool material systems—would require recalibrating material parameters to maintain prediction accuracy.

It is important to note that, while indirect validation has been achieved through comparisons of macroscopic cutting forces, chip morphology, and surface-layer hardness, direct experimental verification of the predicted subsurface dislocation-structure gradients and specific phase transformation products awaits further detailed investigation. In summary, the optimization recommendations presented in this section offer guidance for designing precision-machining processes grounded in the targeted control of microstructural outcomes.

## 5. Conclusions

This research combined analytical and finite element modeling to study the minimum cutting thickness in the turning of 304 stainless steel. Utilizing dislocation density theory, it also enabled the simulation and prediction of microstructure evolution within the machined subsurface layer, analyzing how different tool geometries affect material separation and microstructural changes. The principal conclusions are as follows:

(1) The minimum cutting thickness for 304 stainless steel was determined to be 0.275 times the tool nose radius. While both the minimum cutting thickness and the DMZ area grow with an increasing nose radius, the ratio between the minimum cutting thickness and the nose radius remains stable within 0.25 to 0.30, irrespective of the radius size.
(2) The asymmetric morphology of the cutting edge, characterized by the K-factor, significantly affects the minimum uncut chip thickness by modifying the material separation mode. Investigation reveals a non-monotonic variation in the minimum uncut chip thickness with the K-factor (ranging from 0.5 to 1.5): it initially increases, reaches a maximum under the symmetric edge condition (K = 1.0), and then decreases. This demonstrates that a symmetric edge configuration fosters the development of a more stable and distinct DMZ, consequently delaying the material separation point. In contrast, asymmetric edges (e.g., waterfall or trumpet types) alter the local effective rake angle and stress distribution, thereby facilitating earlier material shear or inducing lateral flow, thereby lowering the critical thickness required for stable chip formation. Therefore, the minimum uncut chip thickness serves as a crucial quantitative indicator of how variations in cutting-edge micro-geometry influence material separation behavior.
(3) The cross-scale predictive model grounded in dislocation density theory has demonstrated high reliability. By successfully integrating a developed user subroutine into the finite element framework, the model enables concurrent prediction of the distribution of dislocation density, the extent of grain refinement, and microhardness gradients within the machined subsurface. Across various cutting speeds, the simulated micro-hardness values show close agreement with experimental measurements in both their variation trends and absolute magnitudes. Beyond replicating the trend of increasing work-hardened layer depth and surface hardness with elevated cutting speed, the model also captures the associated attenuation in the rate of hardness increase—a phenomenon attributed to thermal softening. These results validate the model’s effectiveness in coupling thermo-mechanical effects and its potential for engineering applications in predicting surface integrity.
(4) The parameters defining cutting-edge micro-geometry exert a systematic, yet differentiated, influence on microstructural evolution. Quantitative analysis identifies the tool nose radius as the predominant factor governing the severity of plastic deformation in the surface layer. An increased radius markedly intensifies extrusion and ploughing, resulting in a higher peak dislocation density, more pronounced grain refinement, elevated microhardness, and a substantial increase in the altered layer’s depth (from 56 μm to 147 μm). A reduction in the tool rake angle similarly enhances plastic deformation, leading to increased dislocation density and hardness, alongside decreased grain size, with this effect particularly pronounced in the hostile rake angle regime. The influence of the K-factor on the microstructure mirrors its non-monotonic impact on the minimum uncut chip thickness. In summary, the hierarchical order of influence of these edge parameters on the evolution of the machined surface microstructure is tool nose radius > tool rake angle > cutting edge asymmetry. These established correlations provide direct theoretical guidance for the targeted design of tool edge geometry and the optimization of process parameters to meet specific surface integrity objectives.

## Figures and Tables

**Table 1:** Material properties of 304 stainless steel [29].

Parameter	Value
Density (kg/m^3^)	7950
Specific heat (J/kg/K)	500
Thermal conductivity (W/m/K)	16.2
Thermal expansion coefficient (1/K)	16 × 10^−6^
Young’s modulus (GPa)	193
Poisson’s ratio	0.28
Yield strength (MPa)	235

**Table 2:** J-C constitutive model parameters for 304 stainless steel [33].

A	B	n	C	m	$\dot{\varepsilon }$	$T_{m}$	$T_{r}$
452	694	0.311	0.0067	0.996	0.001	1673 K	273 K

**Table 3:** Orthogonal cutting experimental conditions and results.

Case	v (m/min)	f (mm/rev)	ap (mm)	Main Cutting Foce (N)	Radial Thrust Force (N)
1	90	0.05	1	159	58
2	90	0.1	1	264	96
3	90	0.15	1	357	117
4	150	0.1	1	239	81
5	210	0.1	1	225	75

## Data Availability

The original contributions presented in this study are included in the article. Further inquiries can be directed to the corresponding author.

## Abbreviations

DMZ	Dead metal zone
FEM	Finite element method
ALE	Arbitrary Lagrangian-Eulerian
J-C	Johnson–Cook
CNC	Computerized numerical control
EXP	Experimental
MQL	Minimum quantity lubrication
$h_{Dmin}$	Minimum cutting thickness
$F_{ex}$	Normal force component
$F_{ez}$	Tangential force component
${\;p}_{e}$	Normal stress
r	Tool edge radius
μ	Friction coefficient
θ	Central angle
$F_{px}$	Principal cutting force
$F_{pz}$	Cutting resistance
$\tau_{s}$	Material shear strength
w	Tool width
$\beta_{p}$	Friction angle
$\gamma_{o}$	Tool rake angle
ϕ	Shear angle
ε	Equivalent plastic strain
$\dot{\varepsilon }$	Equivalent plastic strain rate
${\dot{\varepsilon }}_{0}$	Reference strain rate
$T_{r}$	Room temperature
$T_{m}$	Melting temperature
T	Instantaneous deformation temperature
A, B, C, n and m	J-C constitutive model parameters
D1~D5	Johnson–Cook damage parameters
v	Cutting speed
f	Feed rate
ap	Back engagement of cutting edge
$\alpha^{*}$, $\beta^{*}$, and $k_{0}$	Material parameters of dislocation evolution
${\dot{\gamma }}_{0}$	Reference shear strain rate
b	Burgers vector magnitude
n	Temperature sensitivity parameter
$f_{0}$ and $f_{\infty }$	Initial and saturated volume fractions
$\gamma^{r}$	Shear strain
${\bar{\gamma }}^{r}$	Reference plastic strain
